# Improving integrative searching of systems chemical biology data using semantic annotation

**DOI:** 10.1186/1758-2946-4-6

**Published:** 2012-03-08

**Authors:** Bin Chen, Ying Ding, David J Wild

**Affiliations:** 1 School of Informatics and Computing, Indiana University, Bloomington, IN, USA; 2 School of Library and Information Science, Indiana University, Bloomington, IN, USA

## Abstract

**Background:**

Systems chemical biology and chemogenomics are considered critical, integrative disciplines in modern biomedical research, but require data mining of large, integrated, heterogeneous datasets from chemistry and biology. We previously developed an RDF-based resource called Chem2Bio2RDF that enabled querying of such data using the SPARQL query language. Whilst this work has proved useful in its own right as one of the first major resources in these disciplines, its utility could be greatly improved by the application of an ontology for annotation of the nodes and edges in the RDF graph, enabling a much richer range of semantic queries to be issued.

**Results:**

We developed a generalized chemogenomics and systems chemical biology OWL ontology called Chem2Bio2OWL that describes the semantics of chemical compounds, drugs, protein targets, pathways, genes, diseases and side-effects, and the relationships between them. The ontology also includes data provenance. We used it to annotate our Chem2Bio2RDF dataset, making it a rich semantic resource. Through a series of scientific case studies we demonstrate how this (i) simplifies the process of building SPARQL queries, (ii) enables useful new kinds of queries on the data and (iii) makes possible intelligent reasoning and semantic graph mining in chemogenomics and systems chemical biology.

**Availability:**

Chem2Bio2OWL is available at http://chem2bio2rdf.org/owl. The document is available at http://chem2bio2owl.wikispaces.com.

## Background

Recent efforts [1-3] in the Semantic web have involved conversion of various chemical and biological data sources into semantic formats (e.g., RDF, OWL) and linked them into very large networks. The number of bubbles in Linked Open Data (LOD) [4] has expanded rapidly from 12 in 2007 to 203 in 2010. This richly linked data allows answering of complex scientific questions using the SPARQL query language [5], finding paths among objects [6], and ranking associations of different entities [7,8]. Our previous work on Chem2Bio2RDF [3] offers a framework to data mine systems chemical biology and chemogenomics data, as exemplified by the examples given in our paper: compound selection in polypharmacology, multiple pathway inhibitor identification and adverse drug reaction - pathway mapping. However, without an ontology and associated annotation, the utility of the resource is semantically very limited - for example results cannot be refined based on criteria of the type of relationship between entities (e.g., activation or inhibition between compound and protein). Even when it is possible to create a SPARQL query, the lack of ontology increases the complexity of the query: for example, when searching for the targets of a given drug, we have to specify in the SPARQL exactly which databases are to be searched and how to combine the results. SPARQL construction thus requires understanding of the RDF schema of each data source, greatly increasing its complexity. The owl:sameAs (or seeAlso) predicate is used as the primary method for linking multiple data sources sharing common information. Such database level integration does not satisfy our requirement that a query is constructible in a natural and intuitive manner.

An ontology is a formal description of knowledge as a set of concepts within a domain, and the relationships between those concepts. Web Ontology Language (OWL) is a language for making these descriptions designed for use within Semantic Web. A variety of ontologies in the life sciences have been developed. Gene Ontology (GO) [9] is arguably the most widely used ontology in life sciences. It aims to formalize the representation of information about biological processes, molecular functions, and cellular components across multiple organisms. As a part of GO project, the Sequence Ontology consists of a set of terms and relationships used to describe the features and attributes of biological sequence [10]. PRotein Ontology (PRO) describes the relationships of proteins and protein evolutionary families and represents the multiple protein forms of a gene locus [11]. Structurally similar to GO, ChEBI provides ontologies of chemical compounds of biological interest based on their chemical structural and functional features [12]. Disease Ontology (DO) [13] is an open source ontology for the integration of human disease data. Terms in DO are well defined, using standard references and linked to well-established, well-adopted terminologies used in other disease presentations such as MeSH, OMIM, and UMLS. Other domain-specific ontologies have also been developed, including pharmacogenomics [14], ligand protein interaction [15,16], Disease-Drug Correlation Ontology (DDCO) [17], biological pathways (BioPAX) [18], Translational Medicine Ontology [19] and neuromedicine (SWAN) [20]. Particularly, several ontologies have been developed recently to formalize chemical biology experiments and provide guidance for data annotation. For example, the Minimum Information About a Bioactive Entity (MIABE) [21] aims to provide guidelines for reporting bioactive entities explicitly. BioAssay Ontology [22] is developed to standardize the description of HTS experiments and screening results. DDI [23] and OBI [24] present integrative and semantic frameworks in drug discovery investigation and biomedical investigations respectively. A number of upper ontologies such as Basic Formal Ontology (BFO) [25] are developed to support domain ontology building as well. Many of the ontologies are deposited in the OBO foundry [26] or NCBO BioPortal [27], for public access. Using ontologies to integrate data and reason has been widely practiced in life sciences. Baitaluk and Ponomarenko built IntegromeDB to semantically integrate over 100 experimental and computational data sources relating to genomics, transcriptomics, genetics, and functional and interaction data concerning gene transcriptional regulation in eukaryotes and prokaryotes [28]. Holford et al. created logical rules using Semantic Web Rule Language to answer research questions pertaining to pseudogenes [29].

Systems Chemical Biology [30] (and its sub-discipline of chemogenomics) is a new discipline studying how chemicals interact with the whole biological systems, the data of which cover a wide range of entities (compounds, drugs, proteins, genes, diseases, side-effects, pathways, and so on) and various relations between entities such as drug-drug interaction, drug-target interaction, protein-protein interaction and so on. Within this field, chemogenomics is specifically concerned with ways of modeling the relationships between chemical compounds, genes and protein targets. Until now, no systematic ontologies have been developed for chemogenomics, or for the parent field of Systems Chemical Biology. In this work, we describe the creation of such an ontology that covers chemogenomics and the entities of Systems Chemical Biology described above, as defined by the scope of our Chem2Bio2RDF data resource and demonstrate its usage as a knowledge base for study.

## Methods

**Figure 1:**
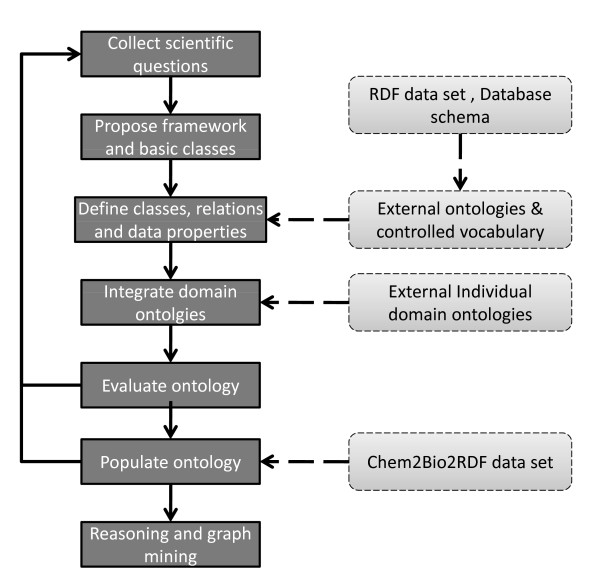
**Workflow for the development of Chem2Bio2OWL**.

The process we used to develop Chem2Bio2OWL is shown in Figure 1. In particular, our ontology was driven by use-case queries that can be found on the Chem2Bio2OWL website, which are difficult or impossible to answer without an ontology. Some examples (semantic terms highlighted in boldface) are:

1. What are the **protein targets **of the **drug Troglitazone**?

2. Find **PPARG inhibitors **with **molecular weight **less than 500.

3. Which **pathways **will be **affected **by **Troglitazone**?

4. Find all **bioassays **that **contain **activity data for a particular **target**.

5. What **liver-expressed proteins **can a given **compound interact **with?

6. Which **proteins **are able to **interact **with **protein PPARG in vivo**?

7. Which **drugs **are used to **treat diabetes **but **withdrawn **from market?

8. Which **assays **test the **activity **of **Troglitazone **against **PPARG **? preferably give the **literature**.

### Classes, relations and data properties

Once we created an initial set of terms derived from use-case queries, we defined a set of primary classes: SmallMolecule, Drug, Protein Target, Disease, SideEffect, Pathway, BioAssay, Literature and Interaction (Table 1) based partially on the BioPAX classes [18]. BioPAX offers a standard, well defined representation of biological pathway data using OWL and it has been widely used in biological data integration [15,31]. We imported the terms from BioPAX and made subsequent extensions based upon our use cases. The primary classes were refined in accordance with current instance data structure. SmallMolecule, Drug and Protein were put under PhysicalEntity. Their relation with Disease and SideEffect were elaborated under Interaction, which is further classified into DrugInducedSideEffect, DrugTreatment, DrugDrugInteraction, ProteinProteinInteraction and ChemicalProteinInteraction. BioAssay and Literature serve as Evidence to support the relations. Pathway was treated as a 'black box' since its instance data is just pathway name. Other than Interaction, we did not intend to further classify other individual major classes.

**Table 1:** Primary classes, their description, sample instance data sources and the number of sample annotated instances.

primary classes	description	sample instance data sources	# of sample instances
SmallMolecule	a small bioactive molecule	PubChem, ChEBI	15509

Drug	a chemical used in the treatment, cure, prevention, or diagnosis of disease	DrugBank, PharmGKB, TTD	6544

Protein	a physical entity consisting of a sequence of amino acids	Uniprot, HGNC, GOA	12242

BioAssay	an experiment to measure the effects of some substance on target, cell or a living organism	PubChem BioAssay, ChEMBL, BindingDB, PDSP	26861

Disease	any condition that causes pain, dysfunction, distress or social problems	OMIM, DO	8724

SideEffect	undesired effect from a medicine	SIDER	1385

Literature	a scientific article	Medline	28392

Pathway	a set or series of a biological interactions	KEGG, Reactome	347

Interaction			

DrugDrug-Interaction	a drug affects the activity of another drug	DrugBank, DCDB	9690

ProtienProtien-Interaction	two or more proteins bind together	HPRD, DIP, BioGrid	54345

DrugInduced-SideEffect	a drug interaction that results in side effect	SIDER	61102

DrugTreatment	the use of drug to treat disease	Diseasome	812

ChemicalProtein-Interaction	genomic response to chemical compounds	ChEMBL, BindingDB, PDSP Ki, TTD, BindingMOAD, DrugBank, CTD, MATADOR, Array-Express, KEGG	47282

Data sources were described in [32]

After major classes were determined, some utility classes were created to help present primary classes, of which a single class is insufficient to present the hierarchical behavior. For instance, ChemicalStructure consisting of structure format and structure representation is considered as a utility class to present the structure of a small molecule. A small molecule may have multiple structure representations, thus there are several instances of ChemicalStructure relating to the small molecule. Without the bearer small molecule, the instance of ChemicalStructure is meaningless.

**Figure 2:**
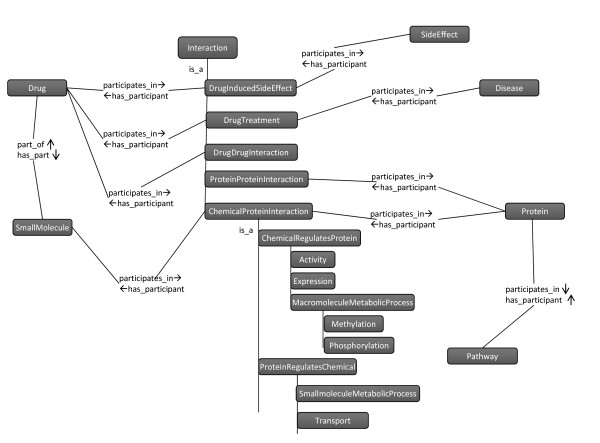
**Overview of Chem2Bio2OWL**. Only part of classes (presented as nodes) and their relations (presented as edges) are visualized. Some classes in ChemicalProteinInteraction are ignored due to the limited space.

The relations between entities which associate with properties (or contexts) such as experimental conditions and references were separated out as individual classes, and were placed under Interaction; otherwise, they were presented as object properties. Relational Ontology (RO) [33] was imported to help present basic relations. For example, ProteinProteinInteraction not only covers the binary relation between two proteins, but also affiliates its experimental conditions (e.g., organism and interaction type). Protein serves as a participant in that interaction. Similarly, Chemical and Protein serve as participants in the ChemicalProteinInteraction, which includes other information such as the strength of interaction. Figure 2 shows major classes and their relations.

Data properties appeared in the original database sources were not fully covered, instead, only the important ones related to our purpose (chemogenomics and systems chemical biology). This simplifies the ontology without losing essential knowledge. The terms including data property name, class name and relation name were manually mapped to terms in relevant ontologies in the OBO and NCBO BioPortal, and the terms in the existing ontologies are preferred if multiple terms happened. For example, for a chemical formula we chose chemicalFormula as this term is used in BioPAX. In addition, the term must conform to our name convention. If there were multiple results or no results at all, we would use the terms from primary data bases. A table was created to map data source terms to the standardized and later was applied to annotate instances. The properties of class, object and data property were further edited in protégé [34].

### Chemogenomic interactions

**Figure 3:**
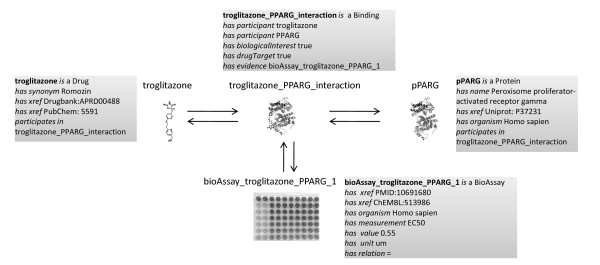
**Ontological representation of Troglitazone, PPARG and their binding association tested in a bioassay experiment**. The real data are available in Chem2Bio2RDF website.

Classification of chemogenomic interactions (compound-protein or drug-target) is extremely important and yet complicated [35]. We consider the interaction from two aspects: 1) how chemicals do with proteins (called ChemicalRegulatesProtein) 2) how proteins do with chemicals (called ProteinRegulatesChemical). ChemicalRegulatesProtein further includes regulation of protein activity, expression of protein, post-modification of protein and so on. ProteinRegulatesChemical includes catalysis of chemical, transportation of chemical and so on. Interaction types described in the Comparative Toxicogenomics Dataset (CTD) [36] were used as a basis for relational terms, being further developed by the addition of new interaction terms such as activation and inhibition. The terms were mapped to GO if exist. In total, 61 interaction classes were created. The experiment to examine the interaction was presented in BioAssay class. The BioAssay outcome includes measurement (e.g., EC50, IC50, Ki, Kd), value, unit (e.g., um, nm) and relation (e.g., ≤, ≥, =). Figure 3 shows how these terms are used in a small network containing drug Troglitazone, gene PPAR-Gamma (PPARG), their interaction and the associated experiment. Only one entry in Chem2Bio2RDF is presented in this figure, there are 43 entries recording this interaction in 6 chemogenomics databases (available at http://cheminfov.informatics.indiana.edu/rest/Chem2Bio2RDF/cid_gene/5591:PPARG).

### Implementation

**Figure 4:**
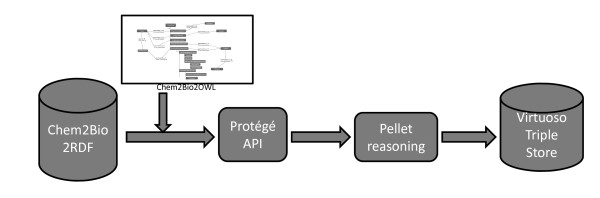
**Workflow for ontology population**.

Figure 4 shows the data integration workflow we used to populate the ontology. Customized Java scripts along with the OWL API Java package [37] were used to automate the annotation of Chem2Bio2RDF data using Chem2Bio2OWL. Pellet reasoning [38] was then applied to reason new relations. The annotated data plus new relations were uploaded to the Virtuoso triple store [39] for querying. Efforts were made to cope with data redundancy, inconsistence and provenance. Data redundancy is originated from the homogeneity of data source of the objects. Chemical compounds for example were presented as various formats (e.g., SMILES, InChi, MOL, etc.) and many data sources have their own identifiers to present compounds. The URI of individual instance in Chem2Bio2OWL is based on the primary data source ID or fake ID if primary ID is unavailable. PubChem as the largest public compound hub is considered as the primary source for chemicals. Its identifier Compound ID (CID) was used to identify compounds (e.g., http://chem2bio2rdf.org/chem2bio2owl#compound5591). The compounds with unknown CIDs were assigned CIDs by searching PubChem using InChi, a universal structure representation. A fake CID was assigned if the compound did not exist in PubChem. Drug, protein and side effect are using DrugBank ID, UNIPROT entry name, and UMLS ID as primary IDs. Pathway name is used as pathway identifier. Diseases can be presented as MESH, OMIM ID, UMLS or free text, but no universal disease identifier has been agreed to present them. Since the Disease Ontology [13] has already mapped terms to various public disease identifiers, we adopted Disease Ontology ID as primary ID. The free texts occurred in TTD, Diseasome and other sources were mapped to disease ontology using string matching algorithms.

Maintaining data provenance (i.e. its source and history) is useful for data validation, confidence weighting and to facilitate data update and maintenance. The class UnificationXref defines a reference to an entity in an external resource that has the same biological identity as the referring entity. Its data properties *DB *and *ID *present the name of external source and the related identifier respectively; *comments *is used to put additional information such as why, who, how and how if needed. For example, compound5591 has ID 5591 in PubChem and ID 9753 in ChEBI, they are represented using class UnificationXref. For some assertions (e.g., interaction), PublicationXref is applied to record the original paper reporting the assertion.

Table 1 shows the statistics of sample instances of primary classes as well as sample primary data sources. The total number of triples is 3,084,836, and it increases to 4,411,817 after reasoning. They were later used for evaluation and are available at Chem2Bio2OWL web site.

## Results

Other than the cases studied before [3,32], we applied the annotated data to answer various questions which are detailed on our website. 20 SPARQLs are available at our website. Here we discuss a few examples.

### Drug related target identification

Identification of potential targets for drugs is important for discovering new therapeutic applications as well as identifying potential undesirable side-effects ("off-target interactions"). These kinds of interactions are described in different ways in many different Chem2Bio2RDF datasets: PubChem BioAssay, ChEMBL and BindingDB contain binding experiments; PharmGKB contains genetic variations upon drug response; CTD and Array Express contain expression data; KEGG contains interactions in pathways. To answer the question: "*What are the possible targets of drug (e.g., Troglitazone)? *" previously required a complex SPARQL query explicitly referencing each data set individually [32]. The following SPARQL presents the searching of two chemogenomics database:

PREFIX compound: <http://chem2bio2rdf.org/pubchem/resource/>

PREFIX bindingdb: <http://chem2bio2rdf.org/bindingdb/resource/>

PREFIX drugbank: <http://chem2bio2rdf.org/drugbank/resource/>

PREFIX uniprot: <http://chem2bio2rdf.org/uniprot/resource/>

SELECT ?uniprot_id

FROM <http://chem2bio2rdf.org/pubchem>

FROM <http://chem2bio2rdf.org/drugbank>

FROM <http://chem2bio2rdf.org/bindingdb>

FROM <http://chem2bio2rdf.org/uniprot>

WHERE {

{?compound compound:CID ?compound_cid. FILTER (?compound_cid = 5591). *#Troglitazone PubChem CID is 5591*

?chemical bindingdb:cid ?compound.

?target bindingdb:Monomerid ?chemical.

?target bindingdb:ic50_value ?ic50. FILTER (?ic50 < 10000).

?target bindingdb:uniprot ?uniprot .

?uniprot uniprot:uniprot ?uniprot_id .

}

UNION

{?compound compound:CID ?compound_cid . FILTER (?compound_cid = 5591) .

?drug drugbank:CID ?compound .

?target drugbank:DBID ?drug .

?target drugbank:SwissProt_ID ?uniprot .

?uniprot uniprot:uniprot ?uniprot_id .

}

}

GROUP BY ?uniprot_id

The query combines the searching of two databases BindingDB and DrugBank, which have their own RDF structures. BindingDB and DrugBank use Monomerid and DBID as compound identifiers separately, and adopt uniprot and SwissProt_ID as target identifiers. They have to be distinct in the SPARQL. The SPARQL would become more complicated if more chemogenomics datasets were considered. We can now create a 'one step' query that is independent of the data source by virtue of our ontology:

PREFIX c2b2r: <http://chem2bio2rdf.org/chem2bio2rdf.owl#>

PREFIX bp: <http://www.biopax.org/release/biopax-level3.owl#>

PREFIX ro: <http://www.obofoundry.org/ro/ro.owl#>

PREFIX rdfs: <http://www.w3.org/2000/01/rdf-schema#>

PREFIX rdf: <http://www.w3.org/1999/02/22-rdf-syntax-ns#>

select distinct ?target_name

from <http://chem2bio2rdf.org/owl#>

where

{

?chemical rdfs:label "Troglitazone"^^xsd:string;

ro:participates_in ?interaction .

?interaction rdf:type c2b2r:ChemicalProteinInteraction;

ro:has_participant ?target .

?target rdf:type bp:Protein;

rdfs:label ?target_name .

}

The query is interpreted as: *chemical with label Troglitazone participates in an interaction which is a chemical protein interaction, and the interaction has a participant, which is of type protein*.

For Troglitazone, other than its primary target PPARG, we found the activities of 10 targets are associated with the drug, and the gene expression of 22 targets are either up or down regulated under the treatment of Troglitazone. For example, Troglitazone could be metabolized by several cytochrome P450 enzymes (CYP17A1, CYP2C19, CYP2C8, CYP2C9 and CYP3A4) and also could affect the activity of ABCB11 (bile salt export pump), which may account for the liver toxicity problems of Troglitazone [40]. To further explore their interactions, another question might be raised: "*What assays test the activity of Troglitazone against PPARG ?*". After running the SPARQL below, 9 bioassay experiments appeared in 5 articles were fetched. Although all assays show the positive activity of Troglitazone against PPARG, their values are different under different experiments, the detail of which could be further explored via associated references.

{

?interaction bp:evidence ?bioAssay;

?bioAssay rdf:type c2b2r:BioAssay;

c2b2r:description ?bioAssayDescription;

c2b2r:hasOutcome [c2b2r: measurement ?measurement;

c2b2r: relation ?relation;

c2b2r: value ?value;

c2b2r: unit ?unit

] .

optional {?bioAssay bp:xref [c2b2r: title ?title]}

}

Following the steps in the previous query, this query is interpreted as: *this interaction has evidence which is a bioassay; the assay has description and outcome, and has reference if exists*.

### Target inhibitor/activator searching

Pregnane × receptor (NR1I2) is a transcriptional regulator of the expression of xenobiotic metabolism and transporter genes. It has multiple binding sites, accounting for different functions. Its agonists at the ligand-binding domain would trigger up-regulation of genes, increase the metabolism and excretion of therapeutic agents, and cause drug-drug interactions, but its antagonists counteract such interactions [41]. Due to different binding sites, the two types of compounds may be quite different structurally. Using Chem2Bio2OWL, we are able to answer this question: "*Find NR1I2 agonists and remove compounds with weight ≥ 500*". The following SPARQL was used to retrieve 37 agonists. Their structures are quite different with 6 antagonists retrieved from another query, indicating the significance of classifying the ligands.

{

?interaction rdf:type c2b2r:ReceptorAgonistActivity; *#or ReceptorAntagonistActivity for antagonist search*

ro:has_participant ?chemical .

?chemical rdf:type bp:SmallMolecule;

c2b2r:hasPhysicalProperty [c2b2r:molecularWeight ?weight];

bp:structure [bp:structureFormat "openeye_can_smiles"^^xsd:string; bp:structureData ?structureData].

FILTER(?weight < 500).

}

This query is interpreted as: *this interaction is a receptor agonist activity and has participant which is a small molecule; the molecule has physical property weight smaller than 500, as well as structure with openeye_can_smiles format*.

### Thiazolidinedione side effect study

**Figure 5:**
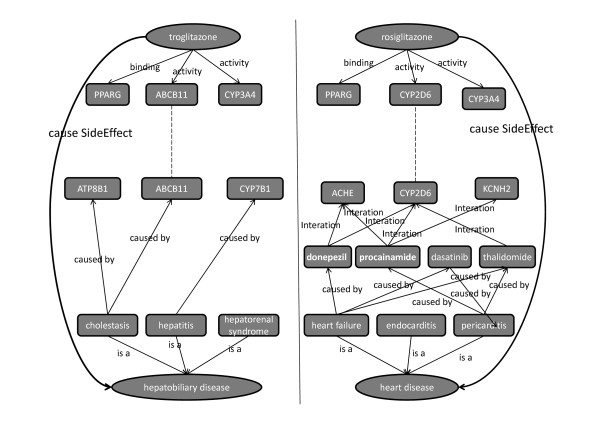
**Thiazolidinediones side effect study: left figure shows the association between Troglitazone and liver toxicity; right figure shows the association between Rosiglitazone and heart disease**.

Thiazolinediones are a class of insulin sensitizing drugs widely used to control diabetes. However, several drugs in the class have suffered from side effects resulting in drug withdrawal (Troglitazone) or restriction (Rosiglitazone). These drugs have a high degree of chemical similarity, but very different side-effects. Troglitazone is associated with an idiosyncratic reaction leading to drug-induced hepatitis or other liver toxicities [42] while Rosiglitazone is associated with an increased risk of myocardial infarction [43]. The systems chemical biology approach has been shown to have the potential to explain drug side effects [44]. Figure 2 illustrates two systems chemical biology approaches to investigate the side effects of Troglitazone and Rosiglitazone. We hypothesize that their related targets might somehow link to disease related genes/proteins, which might explain their side effects. Identification of drug targets and disease related genes/proteins are two major steps. Via the SPARQL for drug related target identification, Troglitazone and Rosiglitazone were found to be associated with to 31 and 48 unique targets respectively via different interactions. Two approaches could be used to find disease related genes/proteins, but the first step would map disease terms into Chem2Bio2OWL disease data. We mapped liver toxicity to hepatobiliary disease in disease ontology which has subclasses such as hepatitis, cholestasis and hepatorenal syndrome, that could be further linked to disease genes in our system (Figure 5). ABCB11 is one of the liver disease related genes and its activity is affected by Troglitazone. ABCB11 involves in the liver bile acid transportation and metabolism (from GO terms for ABCB11). It is not surprising that the change of its activity will result in liver diseases. Similarly, we mapped heart attack to heart disease in the disease ontology that includes heart failure, endocarditis, pericarditis, etc, which are linked to 7 disease genes. However, no overlap between disease genes and Rosiglitazone related targets was found; therefore we then turned to find disease related targets. First, the drugs causing heart disease were searched and their related targets were further identified, grouped, and ranked by the number of their common drugs. The higher ranking indicates the higher possibility linking to the side effect. The top 10 targets are CYP3A4, CYP2C9, ABCB1, CYP1A2, PTGS2, CASP3, CYP2D6, CYP3A5, CYP2C19 and PPARG. The top one CYP3A4 for example is shared by 41 drugs, out of 181 total heart disease related drugs. Some high ranked targets like CYP3A4 also are affected by Troglitazone, nevertheless, the activity of CYP2D6 shared by 24 heart disease related drugs is affected only by Rosiglitazone, it was not found in the Troglitazone related targets. Further literature search indicates that CYP2D6 plays a very important role in cardiovascular disease [45]. Although further experimental evaluation would be preferred, this scenario does demonstrate the usage of Chem2Bio2OWL to investigate systems chemical biology problems.

### Semantic graph mining

**Figure 6:**
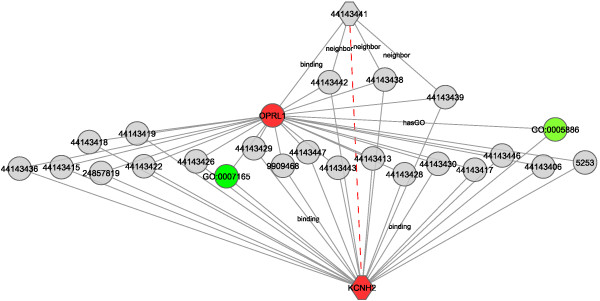
**Paths between compound a benzimidazole analogue (CID:44143441) and target KCNH2 in Chem2Bio2OWL dataset**. Nodes are colored by class and some edges are labeled by interaction type.

Chem2Bio2OWL constructs a complex network with semantic meaning of every component, in which nodes are instances and they are linked by certain semantic relations. This rich set allows the application of various graph mining techniques to discover interesting patterns [46], including simple path-finding between entities. Figure 6 shows how multiple paths link between a benzimidazole analogue (CID:44143441) and the hERG (KCNH2) target. Although there is no reported direct interaction between this compound and this target, the graph visualization suggests their indirect associations. First, three structurally similar compounds (CID:44143442, 44143438 and 44143439) with very high structural similarity to the compound, are able to bind to KCNH2. Second, the target of benzimidazole analogue kappa-type 3 opioid receptor (OPRL1) shares many common compounds with KCNH2, indicating that the compound active in OPRL1 is also possibly active against KCNH2. Third, shared GO terms further manifest the similarity of two targets. Chem2Bio2RDF already is being used in semantic graph mining [8,47,48] to detect various complex relations among drug, target and diseases. The new annotated data greatly increases the utility of this approach.

## Discussion and Conclusions

Many current methods and tools often convert data into RDFs directly from original data bases. They prove useful in their own right to link multiple datasets, but without a formal ontology to model the concepts and their relations, the linked data would not fully demonstrate the capability of semantics, limiting its further usage in data integration and reasoning. In addition, adding evidence and provenance to support assertions is of great importance to keep track of the record, but it has not been well implemented in the current RDFization process. Chem2Bio2OWL provides a high level structure of the entities and relations according to use case queries and instance data structures, and considers evidence and provenance. However, the case driven ontology building process has to make compromises on accuracy to many 'top down' approaches which aims to model domain knowledge oriented from philosophical perspective, nevertheless, our work serves as a local engineering solution to address the urgent needs in this area. The integrative searching of systems chemical biology data can be performed in a very intuitive and efficient way. Other than Chem2Bio2RDF, some public datasets (e.g., Bio2RDF and LODD) could be annotated using Chem2Bio2OWL.

Further efforts should be made to align Chem2Bio2OWL with basic ontology (i.e., BFO and RO) and other Bio-ontologies. For example, the major classes PhysicalEntity and Disease are continuant under BFO, and Pathway, Interaction and BioAssay are occurrent, while other data properties should also be incorporated into the basic ontology so that their usage could be maximized. For example, Utility classes and its subclassess originally serve as helper classes for data integration which are actually modeling artifacts. We did not intend to further model individual major classes, as many of them have their own domain ontology already (e.g., Disease Ontology), which can be incorporated into Chem2Bio2OWL accordingly. Since Chem2Bio2OWL initially was fully based on BioPAX which is originally designed for data integration and data exchange of the biological pathway data and has been widely used, alignment with other basic ontologies needs a collaborative work with BioPAX as well as OBO community.

In summary, we have demonstrated how semantic annotation of systems chemical biology data allows scientifically meaningful, complex queries to be succinctly specified in SPARQL. We present an OWL ontology that was used to annotate our Chem2Bio2RDF set, and is also available for annotation of other integrative chemogenomics and systems chemical biology sets. This ontology was developed through a set of specific scientific use cases, which we believe has made it particularly scientifically relevant. We are currently in the process of aligning this ontology to other widely used ontologies including the Basic Formal Ontology and a variety of biological sets.

## Competing interests

The authors declare that they have no competing interests.

## Authors' contributions

BC, YD and DJW conceived the study, BC carried out the implementation, BC and DJW wrote the manuscript. All contributed to the intellectual evolution of this project. All authors have read and approved the final manuscript.
